# Supplementary material for: Production of Polyhydroxyalkanoates by Two Halophilic Archaeal Isolates from Chott El Jerid Using Inexpensive Carbon Sources
Source: Biomolecules. 2020 Jan 8;10(1):109. doi: 10.3390/biom10010109 (PMC7022696; doi:10.3390/biom10010109)
Supplement: Supplementary file 1 [file biomolecules-10-00109-s001.pdf]

Production of Polyhydroxyalkanoates by Two Halophilic Archaeal Isolates from Chott El Jerid Using Inexpensive Carbon Sources

Manel Ben Abdallah[1], Fatma Karray[1] and Sami Sayadi[2,*]

[1] Laboratory of Environmental Bioprocesses, Centre of Biotechnology of Sfax, BP 1177, Sfax 3018, Tunisia; manelbenabdallah.cbs@gmail.com (M.B.A.); karray.fatma@gmail.com (F.K.)
[2] Center for Sustainable Development, College of Arts and Sciences, Qatar University, Doha 2713, Qatar; sami.sayadi@gmail.com (S.S.)
* Correspondence: sami.sayadi@gmail.com

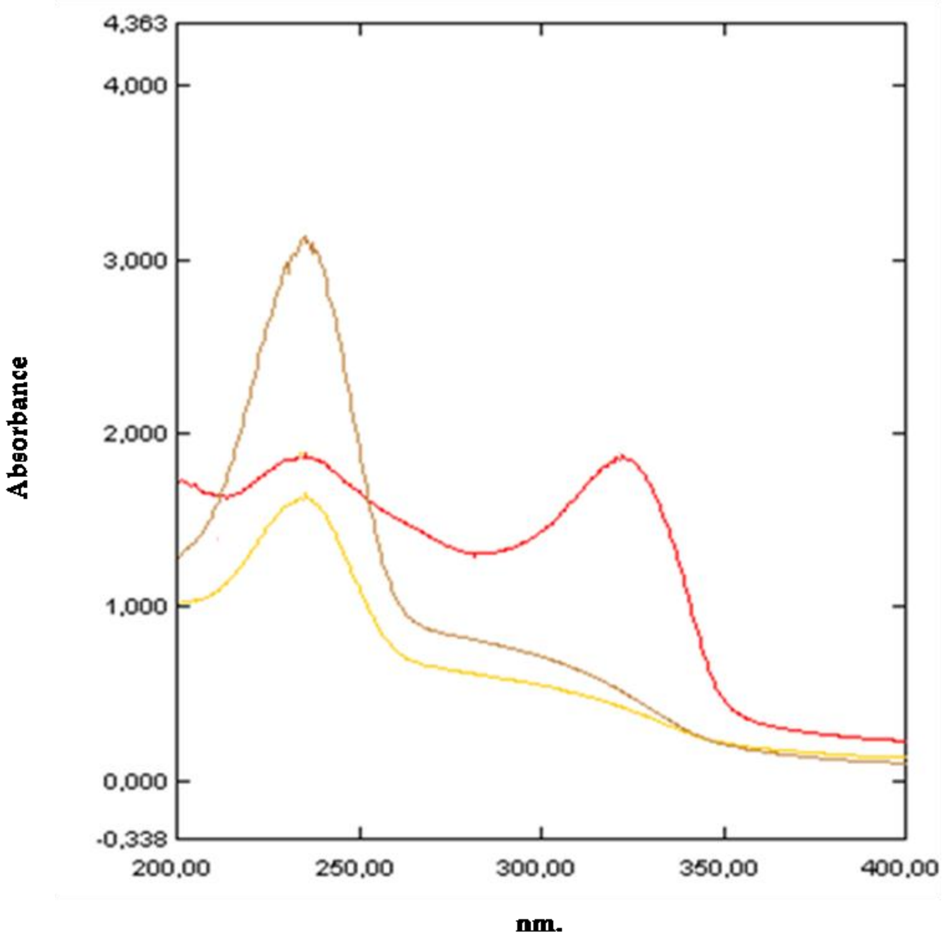

Figure S1: Spectrophotometric scans of crotonic acid of PHA obtained from strains CEJGTEA101 (yellow line) and CEJEA36 (red line) grown in PHA production media supplemented with 2% (w/v) of glucose (best substrate) under optimized conditions of cultivation compared with standard PHB purchased from Sigma-Aldrich (brown line).
